# Numerical investigation of heat and mass transfer in three-dimensional MHD nanoliquid flow with inclined magnetization

**DOI:** 10.1038/s41598-024-51195-4

**Published:** 2024-01-12

**Authors:** Ahmed M. Galal, Fahad M. Alharbi, Mubashar Arshad, Mohammad Mahtab Alam, Thabet Abdeljawad, Qasem M. Al-Mdallal

**Affiliations:** 1 Department of Mechanical Engineering, College of Engineering in Wadi Alddawasir, Prince Sattam Bin Abdulaziz University, Al-Kharj, Saudi Arabia; 2 Production Engineering and Mechanical Design Department, Faculty of Engineering, Mansoura University, P. O 35516, Mansoura, Egypt; 3 Department of Mathematics, Al-Qunfudah University College, Umm Al-Qura University, Mecca, Saudi Arabia; 4 Department of Mathematics, University of Gujrat, Gujrat, 50700 Pakistan; 5 Institute for Numerical and Applied Mathematics, University of Göttingen, 37083 Göttingen, Germany; 6 Department of Mathematics, Abbottabad University of Science & Technology, Abbottabad, 22500, Pakistan; 7 Department of Basic Medical Sciences, College of Applied Medical Science, King Khalid University, 61421 Abha, Saudi Arabia; 8 Department of Mathematics and Sciences, Prince Sultan University, P.O. Box 66833, 11586 Riyadh, Saudi Arabia; 9 Department of Medical Research, China Medical University, Taichung, 40402 Taiwan; 10 Department of Mathematics and Applied Mathematics, Sefako Makgatho Health Sciences University, Garankuwa, 0204 Medusa South Africa; 11 Department of Mathematical Sciences, UAE University, P.O. Box 15551, Al Ain, United Arab Emirates

**Keywords:** Applied mathematics, Fluid dynamics

## Abstract

Heat and mass transfer rate by using nanofluids is a fundamental aspect of numerous industrial processes. Its importance extends to energy efficiency, product quality, safety, and environmental responsibility, making it a key consideration for industries seeking to improve their operations, reduce costs, and meet regulatory requirements. So, the principal objective of this research is to analyze the heat and mass transfer rate for three-dimensional magneto hydrodynamic nanoliquid movement with thermal radiation and chemical reaction over the dual stretchable surface in the existence of an inclined magnetization, and viscous dissipation. The flow is rotating with constant angular speed ${\upomega }^{*}$ about the axis of rotation because such flows occur in the chemical processing industry and the governing equations of motion, energy, and concentration are changed to ODEs by transformation. The complex and highly nonlinear nature of these equations makes them impractical to solve analytically so tackled numerically at MATLAB. The obtained numerical results are validated with literature and presented through graphs and tables. Increasing the Eckert number from $5\le Ec\le 10,$ a higher Nusselt and Sherwood number was noted for the hybrid nanofluid. By changing the angle of inclination $\alpha$*,* the ${Nu}_{x}$ performance is noted at 8% for nanofluid and 33% for hybrid nanofluid. At the same time, ${Sh}_{x}$ performance of 0.5% and 2.0% are observed respectively. Additionally, as the angle of inclination increases the skin friction decreases and the chemical reaction rate increases the mass transmission rate.

## Introduction

In a myriad of industrial applications, ranging from nuclear reactors and automobiles to electronics, the role of fluids is pivotal in enhancing heat transfer rates (HTR). Traditional fluid options like water, oils, and ethylene glycol, however, exhibit limited thermal conductivity. This limitation has spurred extensive research endeavors aimed at elevating HTR for improved efficiency and performance. First, Choi and Eastman^1^ coined the term "nanofluid (NF)" by mixing non-metallic or metallic nanoparticles (NPs) in host fluid which dramatically augments HTR. The characteristics of such fluids depend upon different factors like the shape and size of suspended nanoparticles in host fluid, thermal conduction, etc. Later, many investigators^2–4^ followed his idea and worked on the enhancement of HTR. Similarly, the mixing of two or more nano-sized particles in the host fluid is known as a hybrid nanofluid (HNF). Continuous and different strategies have been adopted to enhance HTR. Jena et al.^5^ explored the recent development in heat transfer (HT) characteristics of NF by considering the non-uniform heat source and inclined magnetization. Parida et al.^6^ computationally discussed the dust particles in water and kerosene-based nanoliquid for HTR. Pattnaik et al.^7,8^ used different NPs like copper, aluminum, gold, and single-wall carbon nanotubes to explain HTR by using water as host fluid over permeable surfaces.

In industrial sectors like aerodynamics, plastic sheet extrusion, continuous metallic plate extrusion, artificial fiber synthesis, and plastic film magnification, the motion of an incompressible fluid over an expanding surface is a frequently observed occurrence. The HTR at the deformable surface plays a substantial role in determining the overall quality of the product in each of these applications. Sakiadis^9^ gave the thought of boundary layer (BL) enhancement during the movement of the surface and attracted researchers' attention. The Sakiadis' problem was expanded by Erickson et al.^10^ who also looked at the causes of puffing or sucking at the moving sheet on HMT in the BL flow. Baag et al.^11^ for exploration of MHD boundary layer flow over porous exponentially SS with a uniform heat source. Nayak et al.^12^ considered the radially stretched sheet for discussion by incorporating the variable magnetic field. Mishra et al.^13^ investigated the HT influence on the MHD movement of micropolar liquid passing from a permeable medium considering a similar heat source. Upreti et al.^14^ used the Casson NF over SS utilizing the Cattaneo-Christov model in stagnation point flow to examine the shape factor. Seini and Makinde^15^ explored the magnetohydrodynamic (MHD) BL flow above the exponentially stretched sheet by considering the chemical reaction (CR). Arshad et al.^16^ considered radiative heat and mass transfer (HMT) for HNF incorporating the inclined magnetic field. Upreti et al.^17^ explored the influence of shape factor on Casson gold-blood nanofluid flow through SS incorporating magnetic effect. Also, considered the impact of Ohmic heating, convective heating, and suction/injection on heat transfer rate. Singh et al.^18^ investigated the influence of Melting and CR on the immobility point flow of a micropolar fluid over a Porous SS Medium. Pandey et al.^19,20^ presented the multiple slip mechanism and volumetric heat generation over porous SS and cone respectively. Sreedevi and Reddy^21^ considered 3D NF flow above SS with radiation and CR for the exploration of thermo-diffusion and Brownian movement. They concluded that enhancing the Deborah number increases the temperature profile.

In recent years, significant research attention has been directed toward Heat and Mass Transfer flows due to their essential nature, prevalent in various engineering and industrial sectors. An example is the extrudate from the die in a melt spinning process. Researchers have investigated multiple flow scenarios, including those involving a stretching surface (SS), chemical reaction (CR), mixed convection, and thermal radiation, to address HMT-related issues. Rao et al.^22^ delved into HMT aspects in the context of a thermally oscillating fluid. Arshad et al.^23,24^ explored HMT by considering thermal radiation and chemical reactions for different types of Newtonian fluids (NFs) and Hybrid Nanofluids (HNFs). Mathur et al.^25^ examined the Darcy–Forchheimer skin coefficients and explored the velocity slip properties of a micropolar NF. Upreti et al.^26^ explored thermodynamics and HT using the Riga plate for the magnetized Casson HNF. They also discussed the entropy generation. Jayavel et al.^27^ provided a discussion on heat transfer analysis and irreversibility in MHD Darcy-Forchheimer movement of Casson HNF flow over wedge and cone. They used nanofluid and hybrid nanofluid for their discussion. Hassan et al.^28^ used the molybdenum di-sulfide NPs to explore the HMT incorporating the non-linear and linear radiation. Hussain et al.^29^ computationally investigated the thermal radiation to find the HTR over a stretchy surface. Different related research^30–38^ in literature can be found. Arshad and Hassan^39^ studied the heat and mass transmission rate in a rotating permeable system using hybrid nanofluids.

Nowadays, chemical reactions, thermal radiation, and the presence of heat source/sink are fundamental components in the study of HMT phenomena. In various engineering applications, the interplay of chemical reactions, thermal radiation, and heat source/sink mechanisms plays a pivotal role in shaping the thermal behavior of systems. Understanding the effects of chemical reactions and thermal radiation, along with heat source/sink interactions, is essential for optimizing processes in fields such as materials science and chemical engineering. Heidary et al.^40^ numerically investigated the magnetic field effect with forced convection in a duct for NF flow. Sheikholeslami and Rokni^41^ gave a review on the simulation for the HT phenomenon of nanoliquid in the existence of a magnetic field. Makinde and Mishra^42^ examined the MHD mixed convection with non-uniform viscosity Blasius flow inserted in a permeable medium incorporating the chemical reaction. Using the CR and heat source, a semi-analytical solution for MHD Jeffery fluid flow is provided by Nisar et al.^43^. Mishra et al.^44^ explained the influence of nonlinear radiation and cross-diffusion effects on the flow of micropolar nanoliquid over a stretching sheet with an exponential heat source. Mehrizi et al.^45^ reported a new analysis of natural convection BL flow with variable wall temperature on a horizontal plate. Reddy et al.^46^ explored the HMT flow of NF at inclined plates with thermal radiation and magnetic fields under enhanced boundary conditions. Nayak et al.^47^ investigated the flow and HTR non-Newtonian fluid with hybrid nanoparticles by employing a magnetic field.

The literature review conducted indicates a notable gap in research, as there has been no investigation into comparing different types of nanofluids over dual stretchable surfaces while factoring in the existence of an inclined magnetic field and accounting for viscous dissipation. Such applications involving nanofluids occur in advanced cooling systems, enhanced heat exchangers, biomedical devices, material processing, renewable energy, etc. Jena et al.^5^ considered only the temperature profile in their study but ignored the concentration profile. Jayavel et al.^27^ made their analysis over the wedge and cone but ignored the inclined magnetization. Pattanaik et al.^7^ examined the uniform heat source and ignored the chemical reaction. Gupta et al.^32^ used the kerosene oil for their investigations over exponentially SS. Arshad and Hassan^39^ deliberated hybrid nanofluids using different NPs. Singh et al.^18^ considered the non-uniform heat source but ignored the thermal radiation. Similarly, Pattanaik et al.^38^, Mohanty et al.^31^, and Parida^6^ presented their research in different aspects but none of these considered the inclined magnetic field. Based on the literature survey conducted above, the novelty of this study aims to provide a comparative analysis of different water-based NFs. These NFs, namely $Cu/{H}_{2}O$; NF, $and Cu-A{l}_{2}{O}_{3}/{H}_{2}O$ HNF, are investigated concerning their HMT rates. This report is prepared for a dual stretching surface placed within a porous medium. Viscous dissipation, thermal radiation, chemical reaction, and most importantly inclined magnetic field are considered because they involve semiconductor manufacturing, solar energy, food processing, etc. The governing equalities are changed into the ODEs by employing a transformation and tackled a MATLAB by BVP-4c algorithm by setting the tolerance ${10}^{-6}$ for solutions. The results are obtained for increasing values of different parameters involved in this research. This comparative analysis serves to provide insights into the central research queries outlined below:

1. How does the rotation parameter affect the velocity, temperature, and concentration profile?
2. What is the influence of increasing behavior of mixed convection, stretching ratio, magnetic force, and inclination angle on velocity and temperature profile?
3. Does the increased thermal radiation and viscous dissipation reduce skin friction and enhance the HTR?
4. How does the chemical reaction affect the concentration profile and Sherwood number?
5. What do we get numerical outcomes for skin frictions along $the x-axis$, $y-axis,$ Nusselt, and Sherwood numbers versus different parameters?

## Problem formulation

**Figure 1:**
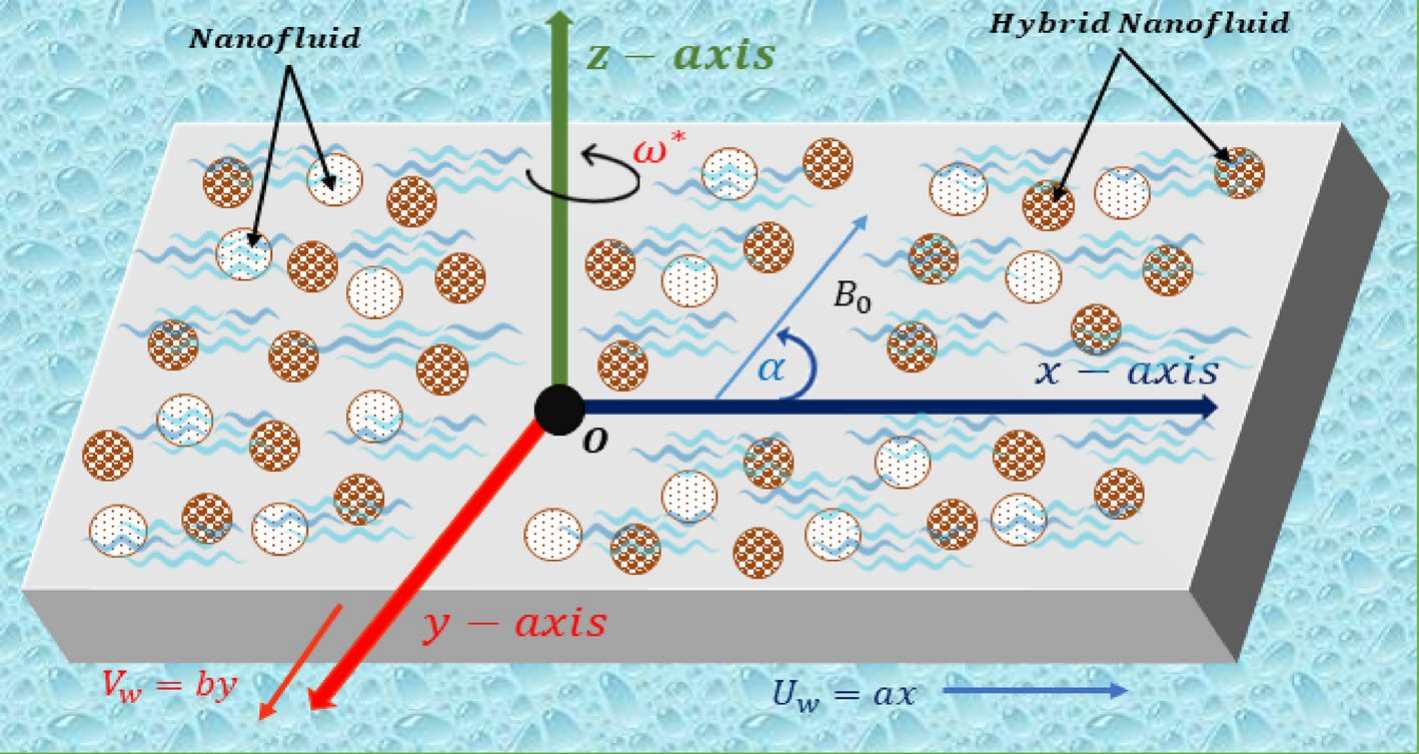
Flow configuration of the problem.

Consider a steady three-dimensional boundary layer, the MHD flow of a fluid over a dual stretching sheet within a porous medium, accompanied by a chemical reaction, thermal radiation, and the existence of a nonuniform heat source. The conceptual representation of the problem, inclusive of the flow design and coordinate system, is presented in Fig. 1. The horizontal direction is represented by the *x*-$axis,$ the upward direction by the* z*-$axis,$ and the y-axis is perpendicular to both axes. The fluid is undergoing a steady rotation at a constant speed denoted as ${\omega }^{*}$ around the* z*-$axis$. The surface is stretching along the* x*-$axis$ with velocity ${U}_{w}=ax$ and along the *y*-$axis$ with velocity ${V}_{w}=by$ as shown. Inclined magnetic field ${B}_{0}$ with angle $\alpha$ from the *x*-$axis$ to the axis of rotation is working. By these assumptions, the flow conservation, momentum along the *x*-*axis*, and *y*-*axis*, temperature and concentration in the existence of joule heating, thermal radiation, and viscous dissipation equation are:

Equation of continuity^48^:

1 $$\frac{\partial \widehat{u}}{\partial x} + \frac{\partial \widehat{v}}{\partial y} + \frac{\partial \widehat{w}}{\partial z} = 0,$$

Momentum equations along the $x$ and $y$ axis^48^:

2 $$\widehat{u}\frac{\partial \widehat{u}}{\partial x} + \widehat{v} \frac{\partial \widehat{u}}{\partial y}+\widehat{w} \frac{\partial \widehat{u}}{\partial z}-2{\omega }^{*}\widehat{v}=\frac{{\mu }_{hnf}}{{\rho }_{hnf}}\left(\frac{{\partial }^{2}\widehat{u}}{{\partial x}^{2}}+\frac{{\partial }^{2}\widehat{u}}{{\partial y}^{2}}+\frac{{\partial }^{2}\widehat{u}}{{\partial z}^{2}}\right)+\frac{{g}^{*}{\left(\rho {B}_{t}\right)}_{hnf}}{{\rho }_{hnf}}\left(T-{T}_{\infty }\right)-\frac{{\sigma }_{hnf}}{{\rho }_{hnf} }{{B}_{0}}^{2}{sin}^{2}\left(\alpha \right)\widehat{u}-\frac{{\mu }_{hnf}}{{\rho }_{hnf} }\frac{\widehat{u}}{{k}_{o}},$$

3 $$\widehat{u}\frac{\partial \widehat{v}}{\partial x} + \widehat{v} \frac{\partial \widehat{v}}{\partial y}+\widehat{w} \frac{\partial \widehat{v}}{\partial z}-2{\omega }^{*}\widehat{u}=\frac{{\mu }_{hnf}}{{\rho }_{hnf}}\left(\frac{{\partial }^{2}\widehat{v}}{{\partial x}^{2}}+\frac{{\partial }^{2}\widehat{v}}{{\partial y}^{2}}+\frac{{\partial }^{2}\widehat{v}}{{\partial z}^{2}}\right)+\frac{{g}^{*}{\left({\rho B}_{t}\right)}_{hnf}}{{\rho }_{hnf}}\left(T-{T}_{\infty }\right)-\frac{{\sigma }_{hnf}}{{\rho }_{hnf} }{{B}_{0}}^{2}{sin}^{2}\left(\alpha \right)\widehat{v}-\frac{{\mu }_{hnf}}{{\rho }_{hnf} }\frac{\widehat{v}}{{k}_{o}},$$

Energy equation without ${q}_{r}$ relation^48,49^:

4 $$\begin{aligned} & \widehat{u}\frac{\partial \widehat{T}}{\partial x} + \widehat{v} \frac{\partial \widehat{T}}{\partial y}+\widehat{w}\frac{\partial \widehat{T}}{\partial z}={\alpha }_{hnf}\left( \frac{{\partial }^{2}\widehat{T}}{{\partial x}^{2}}+\frac{{\partial }^{2}\widehat{T}}{{\partial y}^{2}}+\frac{{\partial }^{2}\widehat{T}}{{\partial z}^{2}}\right)-\frac{1}{{\left({\rho C}_{p}\right)}_{hnf}}\frac{\partial {q}_{r}}{\partial z}\\ &\quad+\frac{{\mu }_{hnf}}{{\left({\rho C}_{p}\right)}_{hnf}}\left[{\left(\frac{\partial \widehat{u}}{\partial z}\right)}^{2}+{\left(\frac{\partial \widehat{v}}{\partial z}\right)}^{2}\right]+\frac{{\sigma }_{hnf}}{{\left({\rho C}_{p}\right)}_{hnf} }{{B}_{0}}^{2}{sin}^{2}\left(\alpha \right)\left[{\widehat{u}}^{2}+{\widehat{v}}^{2}\right]\\ &\quad+\tau \left(\left[{D}_{B}\left\{\frac{\partial \widehat{T}}{\partial x}.\frac{\partial \widehat{C}}{\partial x}+\frac{\partial \widehat{T}}{\partial y}.\frac{\partial \widehat{C}}{\partial y}+\frac{\partial \widehat{T}}{\partial z}.\frac{\partial \widehat{C}}{\partial z}\right\}\right]+\frac{{D}_{T}}{{T}_{\infty }}\left\{{\left( \frac{\partial \widehat{T}}{\partial x}\right)}^{2}+{\left( \frac{\partial \widehat{T}}{\partial y}\right)}^{2}+{\left( \frac{\partial \widehat{T}}{\partial z}\right)}^{2}\right\}\right),\end{aligned}$$

Concentration equation^49^:

5 $$\widehat{u}\frac{\partial \widehat{C}}{\partial x} + \widehat{v} \frac{\partial \widehat{C}}{\partial y}+\widehat{w}\frac{\partial \widehat{C}}{\partial z}={D}_{T}\left( \frac{{\partial }^{2}\widehat{C}}{{\partial x}^{2}}+\frac{{\partial }^{2}\widehat{C}}{{\partial y}^{2}}+\frac{{\partial }^{2}\widehat{C}}{{\partial z}^{2}}\right)+\frac{{D}_{T}}{{D}_{\infty }}\left( \frac{{\partial }^{2}\widehat{T}}{{\partial x}^{2}}+\frac{{\partial }^{2}\widehat{T}}{{\partial y}^{2}}+\frac{{\partial }^{2}\widehat{T}}{{\partial z}^{2}}\right)-{k}_{c}\left(C-{C}_{\infty }\right).$$

The respective boundary conditions^48^ for the current problem are:

6 $$\begin{array}{c}\widehat{u}={U}_{w}=ax, \,\widehat{v}={V}_{w}=by,\, \widehat{w}=0,\, \widehat{T}={\widehat{T}}_{w},\, \widehat{C}={\widehat{C}}_{w},\, at\, z=0 \\ \widehat{u}\to 0, \,\widehat{v}\to 0,\, \widehat{T}\to {\widehat{T}}_{\infty }, \,\widehat{C}\to {\widehat{C}}_{\infty },\, as\, z\to \infty \end{array}$$

Here ${g}^{*}$—gravitational acceleration, ${B}_{0}$—magnetic field, $T$—temperature, $C$—concentration, ${T}_{\infty }$—ambient temperature, ${q}_{r}$—radiative heat flux, $\mu$—dynamic viscosity, $\rho$—density, ${D}_{{\varvec{B}}}$—mass diffusion, ${D}_{T}$—temperature diffusion, $k$—thermal conductivity, ${C}_{p}$—specific heat, $\sigma$—electrical conductivity, ${B}_{t}$—thermal volumetric coefficient, $\alpha$—the angle of inclination, $u,v,w$ are velocity components in $x,y,z$ respectively, the subscript $hnf$ represents the hybrid nanofluid.

The specified boundary conditions for the fluid flow and thermal transport problem outline the physical behavior near and far from the solid surface. At the origin $(z = 0),$ the prescribed velocities ${U}_{w}$ and ${V}_{w}$ denote a stretching wall, while $w=0$ enforces a no stretching. Temperature $(T)$ and concentration $(C)$ conditions ${T}_{w}$ and ${C}_{w}$ at the wall capture heat and mass transfer interactions. As $z\to \infty ,$ the velocity components approach zero $(u\to 0,v\to 0),$ signifying a quiescent state, while temperature and concentration $({T}_{\infty },{ C}_{\infty })$ represent the free-stream values far from the surface. These conditions collectively provide interactions and asymptotic behavior of fluid flow, heat transfer, and mass transport in the given problem.

There are numerous models in literature for characterizing the effective properties of NF and HNF. So, the thermophysical relations of NF and HNF are given in Tables 1 and 2 correspondingly. Table 3 shows the thermophysical values for used NPs and base fluid. Table 4 shows the comparison of literature and present outcomes.

**Table 1:** Thermophysical relations of nanofluid.

Properties	Nanofluid relations^50^
${\text{Density}}$	${\rho }_{nf}=(1-{(\phi }_{1})){\rho }_{f}+{\phi }_{1}{\rho }_{s1}, {G}_{1}=\frac{{\rho }_{nf}}{{\rho }_{f}}$
$\mathrm{Dynamic viscosity}$	${\mu }_{nf}=\frac{{\mu }_{f}}{{[1-\left({\phi }_{1}\right)]}^{5/2}}={K}_{1}$
$\mathrm{Heat capacity}$	${\left(\rho {C}_{p}\right)}_{nf}=\left[1-\left({\phi }_{1}\right)\right]{\left(\rho {c}_{p}\right)}_{f}+{{\phi }_{1}(\rho {c}_{p})}_{s1}, {G}_{2}=\frac{{\left(\rho {C}_{p}\right)}_{nf}}{{\left(\rho {C}_{p}\right)}_{f}}$
$\mathrm{Thermal conductivity}$	$\frac{{k}_{nf}}{{k}_{f}}=\frac{{k}_{s1}+2{k}_{f}-2{\phi }_{1}({k}_{f}-{k}_{s1})}{{k}_{s1}+2{k}_{f}+{\phi }_{1}\times ({k}_{f}-{k}_{s1})}, {G}_{3}=\frac{{k}_{nf}}{{k}_{f}}$
Thermal expansion	${\left(\rho {B}_{t}\right)}_{nf}=(1-{(\phi }_{1})){\rho {B}_{t}}_{f}+{\phi }_{1}{\rho {B}_{t}}_{s1}, {G}_{4}=\frac{{\left(\rho {B}_{t}\right)}_{nf}}{{\left(\rho {B}_{t}\right)}_{f}}$
Electrical conductivity	$\frac{{\sigma }_{nf}}{{\sigma }_{f}}=1+\frac{3\left({\sigma }_{s1}-{\sigma }_{f}\right)}{\left({\sigma }_{s1}+2{\sigma }_{f}\right)-\left({\sigma }_{s1}-{\sigma }_{f}\right){\phi }_{1}}, {G}_{5}=\frac{{\sigma }_{nf}}{{\sigma }_{f}}$

**Table 2:** Thermophysical relations of hybrid nanofluid.

Properties	Hybrid nanofluid relations^39^
${\text{Density}}$	${\rho }_{hnf}=(1-{(\phi }_{1}+{\phi }_{2})){\rho }_{f}+{\phi }_{1}{\rho }_{s1}+{\phi }_{2}{\rho }_{s2}, {H}_{1}=\frac{{\rho }_{hnf}}{{\rho }_{f}}$
$\mathrm{Dynamic viscosity}$	${\mu }_{hnf}=\frac{{\mu }_{f}}{{[1-\left({\phi }_{1}+{\phi }_{2}\right)]}^{5/2}}={K}_{2}$
$\mathrm{Heat capacity}$	${\left(\rho {C}_{p}\right)}_{hnf}=\left[1-\left({\phi }_{1}+{\phi }_{2}\right)\right]{\left(\rho {c}_{p}\right)}_{f}+{{\phi }_{1}(\rho {c}_{p})}_{s1}+{{\phi }_{2}(\rho {c}_{p})}_{s2}, {H}_{2}=\frac{{\left(\rho {C}_{p}\right)}_{hnf}}{{\left(\rho {C}_{p}\right)}_{f}}$
$\mathrm{Thermal conductivity}$	$\frac{{k}_{hnf}}{{k}_{nf}}=\frac{{k}_{s2}+2\times {k}_{nf}-2\times {\phi }_{2}\times \left({k}_{nf}-{k}_{s2}\right)}{{k}_{s2}+2\times {k}_{nf}+{\phi }_{2}\times \left({k}_{nf}-{k}_{s2}\right)},$ *Here *$\frac{{k}_{nf}}{{k}_{f}}=\frac{{k}_{s1}+2\times {k}_{f}-2\times {\phi }_{1}\times ({k}_{f}-{k}_{s1})}{{k}_{s1}+2\times {k}_{f}+{\phi }_{1}\times ({k}_{f}-{k}_{s1})}$*,*${H}_{3}=\frac{{k}_{hnf}}{{k}_{f}}$
Thermal expansion	${\left(\rho {B}_{t}\right)}_{hnf}=(1-{(\phi }_{1}+{\phi }_{2})){\left(\rho {B}_{t}\right)}_{f}+{\phi }_{1}{\left(\rho {B}_{t}\right)}_{s1}+{\phi }_{2}{\left(\rho {B}_{t}\right)}_{s2}, {H}_{4}=\frac{{\left(\rho {B}_{t}\right)}_{hnf}}{{\left(\rho {B}_{t}\right)}_{f}}$
Electrical conductivity	$\frac{{\sigma }_{hnf}}{{\sigma }_{f}}=1+\frac{3\left[\frac{{\sigma }_{s1}{\phi }_{1}-{\sigma }_{s2}{\phi }_{2}}{{\sigma }_{f}}-{(\phi }_{1}+{\phi }_{2})\right]}{\left(2+\frac{{\sigma }_{s1}+{\sigma }_{s2}}{{\sigma }_{f}}\right)-\left[\frac{{\sigma }_{s1}{\phi }_{1}-{\sigma }_{s2}{\phi }_{2}}{{\sigma }_{f}}\right]+{(\phi }_{1}+{\phi }_{2})}, {H}_{5}=\frac{{\sigma }_{hnf}}{{\sigma }_{f}}$

**Table 3:** Thermophysical properties of base fluid^51^ and nanoparticles.

Physical properties	Electrical conductivity	Density	Specific heat	Thermal conductivity	Thermal expansion
${\text{Water}}$	0.05	$997$	$4179$	$0.614$	$21\times {10}^{-5}$
$\mathrm{Copper }\left({{\text{s}}}_{1}\right)$	$5.96\times {10}^{7}$	$8933$	$385$	$400$	$1.67\times {10}^{-5}$
$\mathrm{Aluminum oxide }\left({{\text{s}}}_{2}\right)$	$6.27\times {10}^{-5}$	$3970$	$765$	$40$	$0.85\times {10}^{-5}$

**Table 4:** Comparison of present outcomes with literature.

$\lambda$	Wang^52^		Present outcomes		Nazar et al.^53^	
	${p}^{{\prime}{\prime}}(0)$	${q}^{\prime}(0)$	${p}^{{\prime}{\prime}}\left(0\right)$	${q}^{\prime}(0)$	${p}^{{\prime}{\prime}}(0)$	${q}^{\prime}(0)$
$0.0$	$-1.0$	$0.0$	$-1.013$	$0.0$	$-1.0$	$0.0$
$0.5$	$-1.13$	$-0.51$	$-1.141$	$-0.518$	$-1.13$	$-0.51$
$1.0$	$-1.32$	$-0.83$	$-1.332$	$-0.831$	$-1.32$	$-0.83$
$2.0$	$-1.65$	$-1.28$	$-1.660$	$-1.292$	$-1.65$	$-1.28$

Here $\phi$-volume fraction of NPs, subscripts $f,s,nf,hnf$ are representing fluid, solid nanoparticles, nanofluid and hybrid nanofluid respectively.

The term ${q}_{r}$ in the right side of Eq. (4) presents the heat radiation effect. Utilizing the Rosseland approximation, the radiative heat flux is computed in the following:

7 $${q}_{r}=-\frac{4{\sigma }^{*}}{3{k}_{1}}\frac{{\partial T}^{4}}{\partial z}.$$

Here ${\sigma }^{*}$ represents the Stefan–Boltzmann coefficient and ${k}_{1}$ represents the mean absorption constant. The radiation is optically thick considered. Considering that sufficiently small temperature difference in the flow, the term ${T}^{4}$ by employing the Taylor series as follows:

8 $${T}^{4}={{T}^{4}}_{\infty }+{4{T}^{3}}_{\infty }\left(T-{T}_{\infty }\right)+{6{T}^{2}}_{\infty }{\left(T-{T}_{\infty }\right)}^{2}+\dots$$

Therefore, by abandoning higher-order terms above the first degree in $\left(T-{T}_{\infty }\right),$ we get.

9 $${T}^{4}=4{{T}^{3}}_{\infty }T-3{{T}^{4}}_{\infty }.$$

By using Eqs. (7) and (8)

10 $$\frac{{\partial q}_{r}}{\partial z}=-\frac{16{\sigma }^{*}{{T}_{\infty }}^{3}}{{3k}_{1}}\frac{{\partial }^{2}T}{{\partial z}^{2}},$$

So, Eq. (4) takes the following form:

11 $$\begin{aligned} & \widehat{u}\frac{\partial \widehat{T}}{\partial x} + \widehat{v} \frac{\partial \widehat{T}}{\partial y}+\widehat{w}\frac{\partial \widehat{T}}{\partial z}={\alpha }_{hnf}\left( \frac{{\partial }^{2}\widehat{T}}{{\partial x}^{2}}+\frac{{\partial }^{2}\widehat{T}}{{\partial y}^{2}}+\frac{{\partial }^{2}\widehat{T}}{{\partial z}^{2}}\right)+\frac{16{\sigma }^{*}{{T}_{\infty }}^{3}}{{3k}_{1}{\left({\rho C}_{p}\right)}_{hnf}}\frac{{\partial }^{2}T}{{\partial z}^{2}}\\ & \quad+\frac{{\mu }_{hnf}}{{\left({\rho C}_{p}\right)}_{hnf}}\left[{\left(\frac{\partial \widehat{u}}{\partial z}\right)}^{2}+{\left(\frac{\partial \widehat{v}}{\partial z}\right)}^{2}\right]+\frac{{\sigma }_{hnf}}{{\left({\rho C}_{p}\right)}_{hnf} }{{B}_{0}}^{2}{sin}^{2}\left(\alpha \right)\left[{\widehat{u}}^{2}+{\widehat{v}}^{2}\right]\\ & \quad+\tau \left(\left[{D}_{B}\left\{\frac{\partial \widehat{T}}{\partial x}.\frac{\partial \widehat{C}}{\partial x}+\frac{\partial \widehat{T}}{\partial y}.\frac{\partial \widehat{C}}{\partial y}+\frac{\partial \widehat{T}}{\partial z}.\frac{\partial \widehat{C}}{\partial z}\right\}\right]+\frac{{D}_{T}}{{T}_{\infty }}\left\{{\left( \frac{\partial \widehat{T}}{\partial x}\right)}^{2}+{\left( \frac{\partial \widehat{T}}{\partial y}\right)}^{2}+{\left( \frac{\partial \widehat{T}}{\partial z}\right)}^{2}\right\}\right),\end{aligned}$$

Similarity transformation:

In the present problem, the following similarity transformations are taken to change the PDEs into ODEs.

12 $$\begin{array}{c}\widehat{w}=\sqrt{a{v}_{f} }\left\{p\left(\eta \right)+q\left(\eta \right)\right\}, \,\widehat{v}=ay{q}{\prime}\left(\eta \right),\, \widehat{u}=ax{p}^{\prime}\left(\eta \right), \\ \eta =z\sqrt{\frac{a}{{v}_{f}}}, \,s\left(\eta \right)\left({\widehat{C}}_{o}-{\widehat{C}}_{\infty }\right)=\widehat{C}-{\widehat{C}}_{\infty },\, r\left(\eta \right)\left({\widehat{T}}_{o}-{T}_{\infty }\right)=\widehat{T}-{\widehat{T}}_{\infty }.\end{array}$$

The differentiation is w.r.t $\eta .$ The flow Eq. (1) of mass conversation is satisfied identically by employing Eq. (12). The Eqs. (2,3,5) and Eq. (11) for momentum, energy, and concentration will take the following form after using Eq. (12) with boundary conditions:

13 $${p}^{{\prime}{\prime}{\prime}}\left(\eta \right)={H}_{1}*\left\{{{p}^{\prime}\left(\eta \right)}^{2}*\left({p}^{\prime}\left(\eta \right)+{q}^{\prime}\left(\eta \right)\right)-2* \delta *{q}^{\prime}\left(\eta \right)+Z* {p}^{\prime}\left(\eta \right)-{\epsilon }_{x}*r*{H}_{4}+{M}^{2}*{sin}^{2}\left(\alpha \right)*{p}^{\prime}*{H}_{5}\right\}*{K}_{2}$$

14 $${q}{\mathrm{^{\prime}}\mathrm{^{\prime}}\mathrm{^{\prime}}}\left(\eta \right)={H}_{1}*\left\{{{q}{\mathrm{^{\prime}}}\left(\eta \right)}^{2}\left({p}{\mathrm{^{\prime}}}\left(\eta \right)+{q}{\mathrm{^{\prime}}}\left(\eta \right)\right)-2*\frac{\lambda }{\delta }* {p}{\mathrm{^{\prime}}}\left(\eta \right)+Z* {q}{\mathrm{^{\prime}}}\left(\eta \right)-{\epsilon }_{y}*r*{H}_{4}+{M}^{2}*{sin}^{2}\left(\alpha \right)*{q}{\mathrm{^{\prime}}}*{H}_{5}\right\}*{K}_{2}$$

15 $$\begin{aligned}{r}^{{\prime}{\prime}} & =-{\left(1+\frac{4}{3*{H}_{3}}*\pi \right)}^{-1}\left[Pr*{H}_{2}*{r}^{\prime}\left(p\left(\eta \right)+q\left(\eta \right)\right)+{r}^{\prime}*{s}^{\prime}*{N}_{b}+{{r}^{\prime}}^{2}{N}_{t}\right.\\ & \quad \left.+\left[{K}_{2}*Ec*\left({{p}^{{\prime}{\prime}}}^{2}+{{q}^{{\prime}{\prime}}}^{2}\right)+{H}_{5}*{M}^{2}*{sin}^{2}\left(\alpha \right)*Ec*\left({{p}^{\prime}}^{2}+{{q}^{\prime}}^{2}\right)\right]\right]\end{aligned}$$

16 $${s}{\mathrm{^{\prime}}\mathrm{^{\prime}}}=Sc*\left(\left(p+q\right)*{r}{\mathrm{^{\prime}}}+\frac{{N}_{t}}{{N}_{b}}*{r}{\mathrm{^{\prime}}\mathrm{^{\prime}}}-s*{K}_{c}\right)$$

The non-dimensional quantities ${H}_{1},{ H}_{2}, {H}_{3},{ H}_{4}{, H}_{5},$ and ${K}_{2},$ are hybrid NPs relations (presented in Table 2), and $\lambda ,\delta , Z,\epsilon , M, Pr, \pi , Ec, Sc, {N}_{t},\tau , {N}_{b},$ and ${K}_{c}$ are defined as

17 $$\begin{array}{c}\lambda =\frac{{\omega }^{*}}{a},\delta =\frac{y}{x}, Z=\frac{{\mu }_{hnf}}{{a{\rho }_{hnf}k}_{o}},\epsilon =\frac{\mathrm{ Gr}}{{{Re}_{x}}^{2}}, M= \sqrt{\frac{{\sigma }_{f}{{B}_{0}}^{2}}{a{\rho }_{f}}},\\ Pr=\frac{{v}_{f}}{{k}_{f}}, \pi =\frac{4{\sigma }^{*} {{T}_{\infty }}^{3}}{{k}_{1} {k}_{f}}, Sc= \frac{{D}_{B}}{{v}_{f}},\tau =\frac{{\left(\rho Cp\right)}_{s}}{{\left(\rho Cp\right)}_{f}},\\ Ec=\frac{{{u}_{w}}^{2}}{{\left(\rho {C}_{p}\right)}_{f}}\frac{1}{\left({T}_{w}-{T}_{\infty }\right)},{N}_{t}=\frac{{\left(\rho Cp\right)}_{s}{D}_{B}\left({C}_{w}-{C}_{\infty }\right)}{{T}_{\infty }{\left(\rho Cp\right)}_{f} {v}_{f}},\\ {N}_{b}=\frac{{\left(\rho Cp\right)}_{s}{D}_{B}\left({T}_{w}-{T}_{\infty }\right)}{{\left(\rho Cp\right)}_{f} {v}_{f}},{K}_{c}=\frac{{K}_{r}}{a}\end{array}$$

Here $${\text{Gr}}=\frac{{g}^{*}{\left(\rho {B}_{t}\right)}_{hnf}}{{{v}_{f}}^{2}}\left(T-{T}_{\infty }\right) {x}^{3}, {Re}_{x}=\frac{{u}_{w}}{{v}_{f}}.$$

The modified boundary conditions are presented as follows:

18 $$\begin{array}{c}at \eta =0, \,s=1,\, r=1,\, {q}^{\prime}=\gamma , \,q=0,\, {p}^{\prime}=1, \,p=0, \\ as \eta \to \infty ,\, s\to 0,\, r\to 0, \,{q}^{\prime}\to 0,\, {p}{\prime}\to 0,\end{array}$$

Here $\gamma =\frac{b}{a}$ presents the dimensionless stretching ratio.

**Engineering parameters of interest**:

There are the following most important quantities regarding to engineering perspective.

(A) Skin friction:

The significant surface skin friction along the *x*-$axis$ and *y*-$axis$ are ${Cf}_{x}$, ${Cf}_{y}$ defined as:

19 $${Cf}_{x}=\frac{{\tau }_{zx}}{{\rho }_{f}{{u}_{w}}^{2}}, \,{Cf}_{y}=\frac{{\tau }_{zy}}{{\rho }_{f}{{v}_{w}}^{2}}$$

The ${\tau }_{zx}$ and ${\tau }_{zy}$ indicate shear stress along the stretched wall *x*-$axis$ and *y*-$axis$ which is defined as:

20 $${\tau }_{zx}={\mu }_{hnf}{\left(\frac{\partial u}{\partial z}+\frac{\partial w}{\partial x}\right)}_{z=0} , {\tau }_{zy}={\mu }_{hnf}{\left(\frac{\partial v}{\partial z}+\frac{\partial w}{\partial y}\right)}_{z=0}$$

The dimensionless form of Eq. (20) with the help of Eq. (16) is:

21 $${\left({Re}_{x}\right)}^{1/2} C{f}_{x}=\frac{{\mu }_{hnf}}{{\mu }_{f}} {p}^{{\prime}{\prime}}\left(0\right),\, {\left({Re}_{x}\right)}^{1/2} C{f}_{y}=\frac{{\mu\, }_{hnf}}{{\mu }_{f}} {q}^{{\prime}{\prime}}\left(0\right),$$

(B) Rate of heat and mass transfer:

The HMT rates, expressed through Nusselt and Sherwood numbers are the following:

By utilizing the temperature field, the thermal diffusion rate is characterized by the Nusselt number:

22 $${Nu}_{x}=\frac{x{q}_{w}}{{k}_{f}({T}_{w}-{T}_{\infty })}, \,{q}_{w}=-{k}_{hnf}{\left(\frac{\partial T}{\partial z}\right)}_{z=0}+ {{(q}_{r})}_{w},$$

By using the similarity transformation, the transformed form of Eq. (22) is given below:

23 $${Nu}_{x}=-\left({B}_{3}+\frac{4}{3}\pi \right){r}{\prime}\left(0\right),$$

By utilizing the concentration field, the mass transmission rate is characterized by the Sherwood number:

24 $${Sh}_{x}=\frac{x{q}_{m}}{{D}_{m}\left({C}_{w}-{C}_{\infty }\right)}, {q}_{m}={D}_{m}{\left(\frac{\partial C}{\partial z}\right)}_{z=0}$$

By using the similarity transformation, the transformed form of Eq. (24) is given below:

25 $${Sh}_{x}=-{s}{\prime}\left(0\right).$$

## Numerical solution

**Figure 2:**
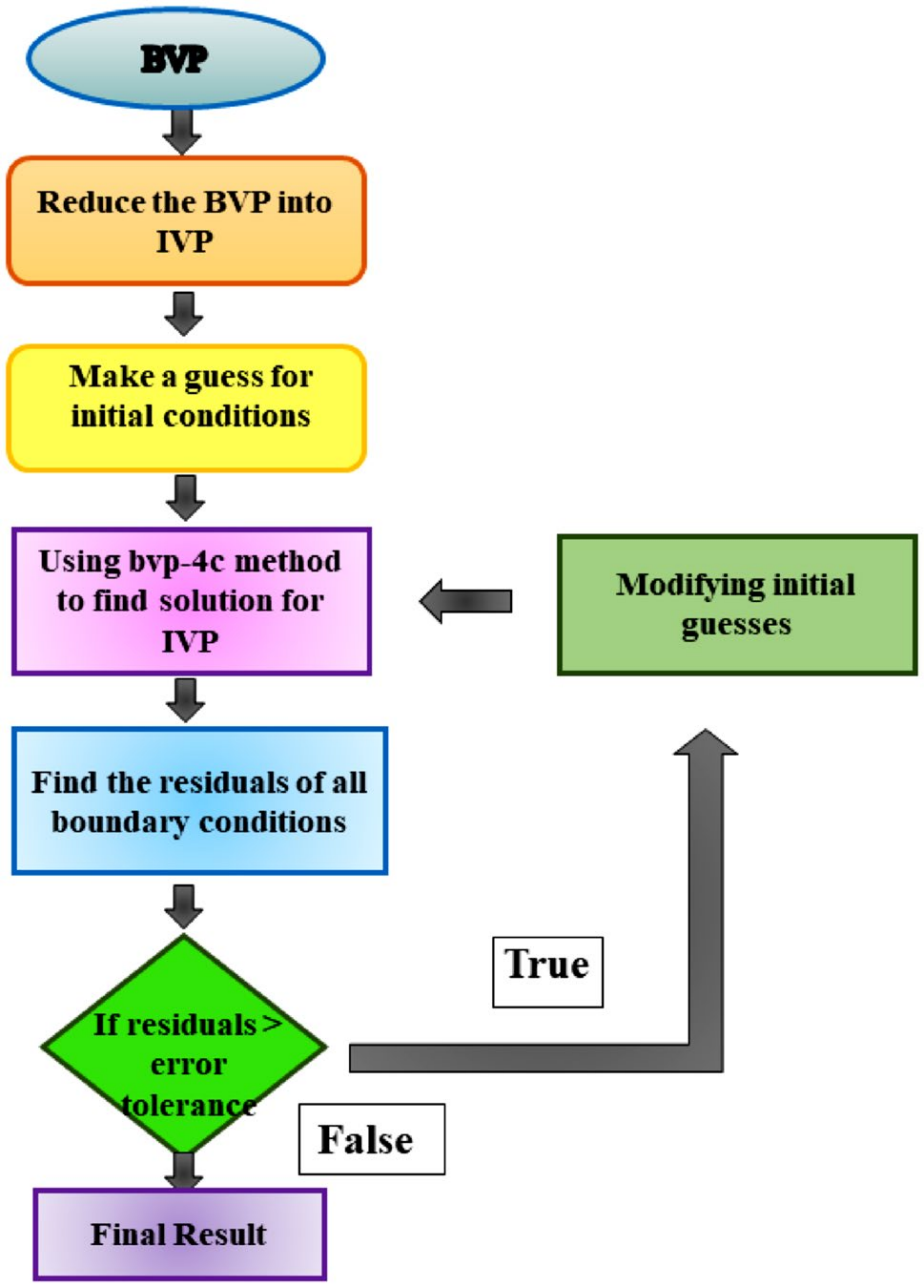
Flow chart for the numerical solution.

**Figure 3:**
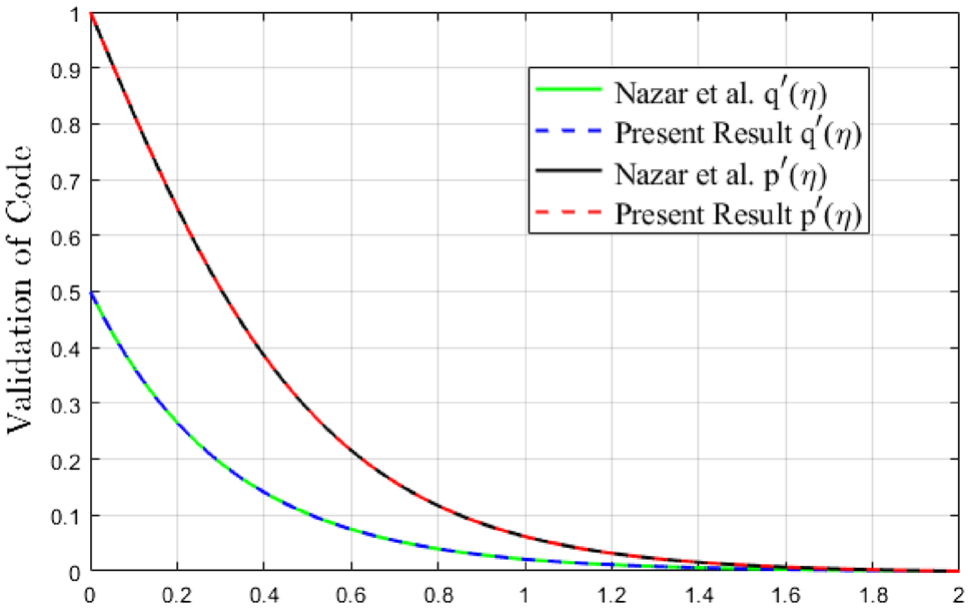
Validation of code by velocity profile ${p}^{\prime}\left(\eta \right)$ and ${q}^{\prime}\left(\eta \right)$.

This section provides the numerical solution methodology as presented in Fig. 2 to get the solution of higher-order nondimensional ODEs. For this purpose, the bvp4c method is used to tackle the boundary value problem by transforming it into an initial value problem. The Fig. 3 validates the code of the present problem. For this purpose, newly defined variables are as follows:

26 $$\begin{gathered} z_{3}{\prime} = p^{\prime\prime\prime}, z_{3} = p^{\prime\prime}, z_{2} = p^{\prime}, z_{1} = p, \hfill \\ z_{6}{\prime} = q^{\prime\prime\prime}, z_{6} = q^{\prime\prime}, z_{5} = q^{\prime}, z_{4} = q, \hfill \\ z_{8}{\prime} = r^{\prime\prime}, z_{8} = r^{\prime}, z_{7} = r, \hfill \\ z_{10}{\prime} = s^{\prime\prime}, z_{10} = s^{\prime}, z_{9} = s, \hfill \\ \end{gathered}$$

The following form of equations is used in MATLAB to get the numerical solution.

27 $${{z}_{1}}^{\prime}={z}_{2}$$

28 $${{z}_{2}}^{\prime}={z}_{3}$$

29 $${{z}_{3}}^{\prime}={H}_{1}*\left\{\left[{{z}_{2}}^{2}*\left({z}_{1}+{z}_{4}\right)\right]-\left[2* \lambda *\delta * {z}_{5}\right]+\left[Z*{z}_{2}\right]-\left[{\epsilon }_{x}*{z}_{7}*{H}_{4}\right]+\left[{M}^{2}*{z}_{2}*{sin}^{2}\left(\alpha \right)*{H}_{5}\right]\right\}*{K}_{2}$$

30 $${{z}_{4}}^{\prime}={z}_{5}$$

31 $${{z}_{5}}^{\prime}{=z}_{6}$$

32 $${{z}_{6}}^{\mathrm{{\prime}}}={H}_{1}*\left\{\left[{{z}_{5}}^{2}*\left({z}_{1}+{z}_{4}\right)\right]-\left[2*\lambda *\frac{1}{\delta }* {z}_{2}\right]+\left[Z*{z}_{5}\right]-\left[{\epsilon }_{y}*{z}_{7}*{H}_{4}\right]+\left[{M}^{2}*{sin}^{2}\left(\alpha \right)*{z}_{5}*{H}_{5}\right]\right\}*{K}_{2}$$

33 $${{z}_{7}}^{\prime}={z}_{8}$$

34 $$\begin{aligned}{{z}_{8}}^{\mathrm{^{\prime}}} & =-{\left(1+\frac{4}{3*{H}_{3}}*\pi \right)}^{-1}\left[Pr*{H}_{2}*{z}_{8}\left({z}_{1}+{z}_{4}\right)+{z}_{8}*{z}_{10}*{N}_{b}+{{z}_{8}}^{2}{N}_{t}\right.\\ & \quad \left.+\left[{K}_{2}*Ec*\left({{z}_{3}}^{2}+{{z}_{6}}^{2}\right)+{H}_{5}*{M}^{2}*{sin}^{2}\left(\alpha \right)*Ec*\left({{z}_{2}}^{2}+{{z}_{5}}^{2}\right)\right]\right]\end{aligned}$$

35 $${{z}_{9}}^{\prime}={z}_{10}$$

36 $${{z}_{10}}^{\mathrm{^{\prime}}}= Sc*\left(\left(\left({z}_{4}+{z}_{1}\right)*{z}_{8}\right)+\left(\left(\frac{{N}_{t}}{{N}_{b}}\right)*{{z}_{8}}^{\mathrm{{\prime}}}\right)-\left({z}_{10}*{K}_{c}\right)\right)$$

The transformed boundary conditions have been modified into the following form:

37 $$\begin{array}{c}at \eta =0,\, {z}_{1}=0,\, {z}_{2}=1,\, {z}_{4}=0, \,{z}_{5}=\gamma , \,{z}_{7}=1, \\ as\, \eta \to \infty ,\, {z}_{2}\to 0,\, {z}_{5}\to 0,\, {z}_{7}\to 0.\end{array}$$

## Results and discussion

The problem is formulated for HNF in the previous section. The authors obtained the results of the current problem separately for both NF and HNF and discussed them physically. The obtained influences of different constitutes by using the bvp4c algorithm at MATLAB are described in the following.

### Velocity profiles

**Figure 4:**
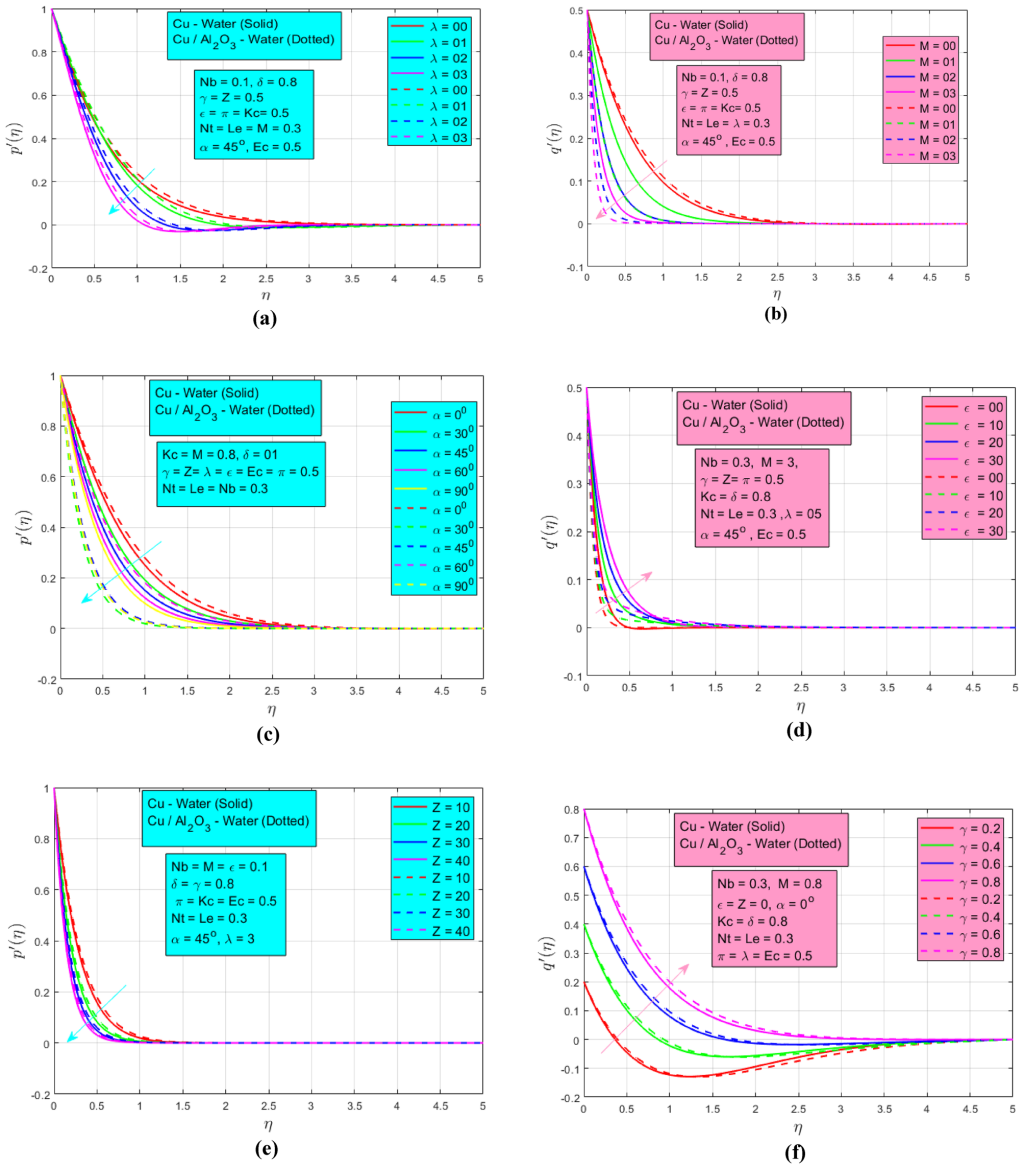
(**a**) Effect of $\lambda$ on velocity ${p}{\prime}(\eta )$. (**b**) Effect of $M$ on velocity ${q}{\prime}(\eta )$. (**c**) Effect of $\alpha$ on velocity ${p}{\prime}(\eta )$. (**d**) Effect of $\epsilon$ on velocity ${q}{\prime}(\eta )$. (**e**) Effect of $Z$ on velocity ${p}{\prime}(\eta )$. (**f**) Effect of $\lambda$ on velocity ${q}{\prime}(\eta )$.

The influences of study parameters on velocities are discussed in this section. The influence of rotation on velocity profile ${p}{\prime}\left(\eta \right)$ when $Nb=0.1,\delta =0.8, \gamma =Z=\epsilon =\pi =Kc=Ec=0.5, Nt=Le=M=0.3, \alpha =45^\circ$ is presented in Fig. 4a. The graph displays the velocity profiles ${p}{\prime}\left(\eta \right),$ demonstrating a decrease in velocity as the rotation parameter $\lambda$ increases for both NF and HNF cases. Initially, at $\lambda =0$ and $\lambda =1,$ there is minimal alteration in the velocity profiles. However, a significant acceleration in the decay of these profiles is observed as the rotation parameter rises to $\lambda =2$ and $\lambda =3.$ The main reason behind this is enhanced viscous dissipation that affects the velocity profile. Figure 4b represents the influence of the magnetic field on velocity ${q}{\prime}\left(\eta \right) \mathrm{when }Nb=0.1,\delta =0.8, \gamma =Z=\epsilon =\pi =Kc=Ec=0.5, Nt=Le=\lambda =0.3, \alpha =45^\circ$. The magnetic parameter has a great influence on the velocity profile. In the non-existence $(M=0)$ of the magnetic field, the momentum BL looks similar but when it upsurges between $1\le M\le 3,$ a big change is noted due to the existing Lorentz force which decreases the fluid motion and consequently momentum BL decay. A wider momentum BL is noted for HNF as associated with NF. This is due to the generation of damping electromagnetic force which restricts the fluid's motion and results in a gradual reduction in velocity profile. Figure 4c depicts the impact of the angle of inclination 0$^\circ \le \alpha \le 90^\circ$ on velocity profile ${p}{\prime}\left(\eta \right)$ $\mathrm{when }Nb=0.1,\delta =0.8, \gamma =Z=\epsilon =\pi =Kc=Ec=0.5, Nt=Le=\lambda =0.3, M=0.8$. The parameter at $\alpha =0$ represents the magnetic force parallel to the $x-axis$ and varies from $\alpha =0^\circ$ to $\alpha =90^\circ$ i.e., parallel to the *y*-$axis.$ As the inclination angle increases, the momentum BL decreases because Lorentz force increases gradually. The extended momentum BL is noted for HNF when $\alpha =30^\circ , 45^\circ$ and 90$^\circ$ whereas NF has smooth BL. A minimum flow for NF is observed when the inclination angle is parallel to the $y-axis$ because greater interaction between the magnetic field and fluid increases flow resistance. The mixed convection parameter $0\le \epsilon \le 30$ has a direct relation with velocity profile ${p}{\prime}\left(\eta \right)$ as presented in Fig. 4d $\mathrm{when }Nb=0.1,\delta =0.8, \gamma =Z=\pi =Kc=Ec=0.5, Nt=Le=\lambda =0.3, \alpha =45^\circ , M=3$ and a higher velocity profile is observed for NF as associated with the HNF. This is owing to the driving forces associated with convection which typically increases the fluid’s velocity. Figure 4e shows that porous medium parameter $Z$ has a negligible decreasing influence on velocity profile ${p}{\prime}\left(\eta \right)$ $\mathrm{when }Nb=0.1,\delta =0.8, \gamma =\epsilon =\pi =Kc=Ec=0.5, Nt=Le=\lambda =0.3, \alpha =45^\circ , M=0.8$. The porosity acts as a flow impediment, and the drag force significantly reduces the velocity of the fluid. The stretching ratio parameter $\gamma$ represents the effects on velocity profile ${q}{\prime}\left(\eta \right)$ in Fig. 4f $\mathrm{when }Nb=0.1,\delta =0.8, \lambda =Z=\epsilon =\pi =Kc=Ec=0.5, Nt=Le=0.3, \alpha =45^\circ , M=0.8$. When the stretching rate along the $x-axis$ increases as compared to the *y*-$axis$, the velocity momentum BL expands for both types of NFs.

### Temperature and concentration profiles

**Figure 5:**
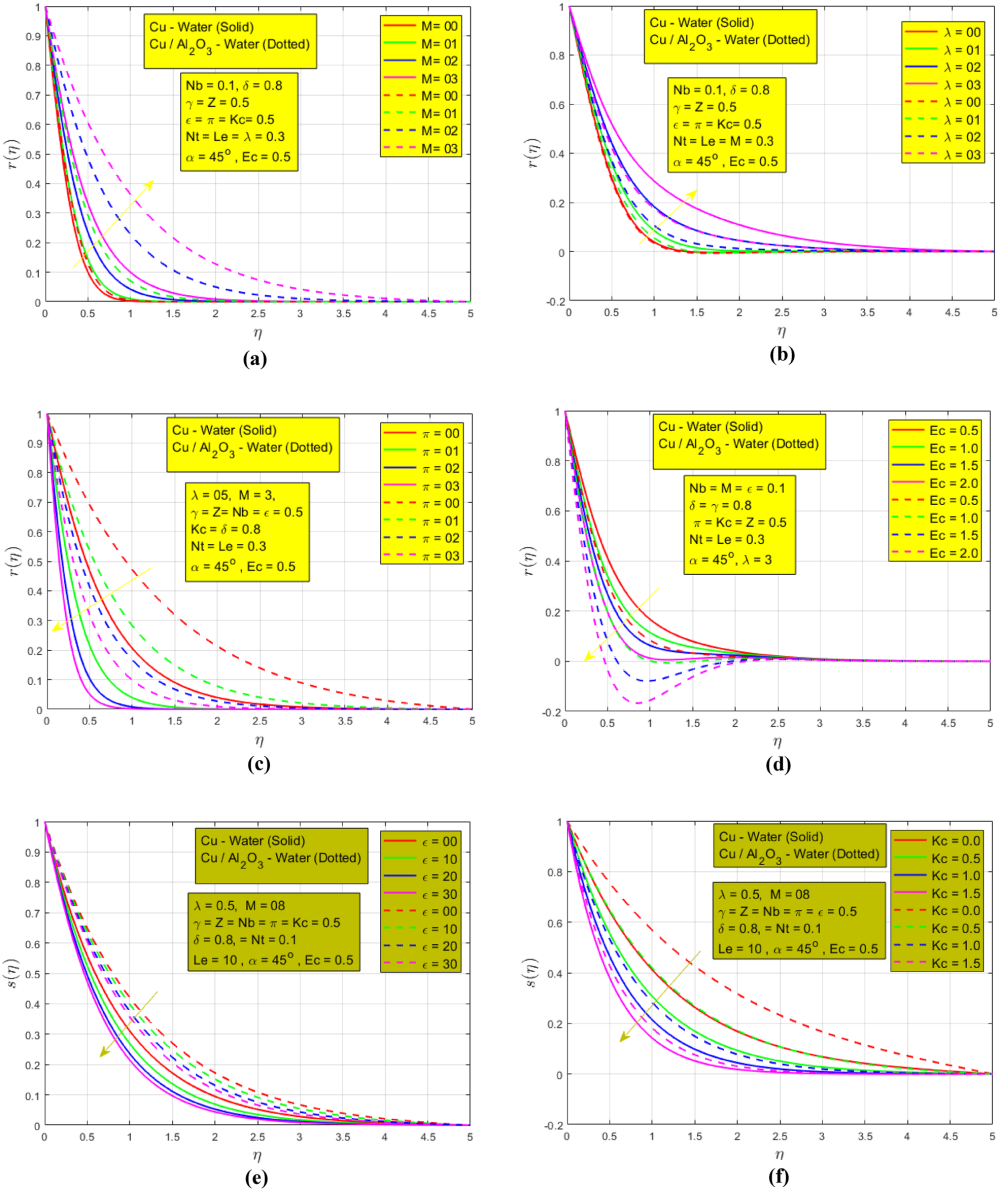

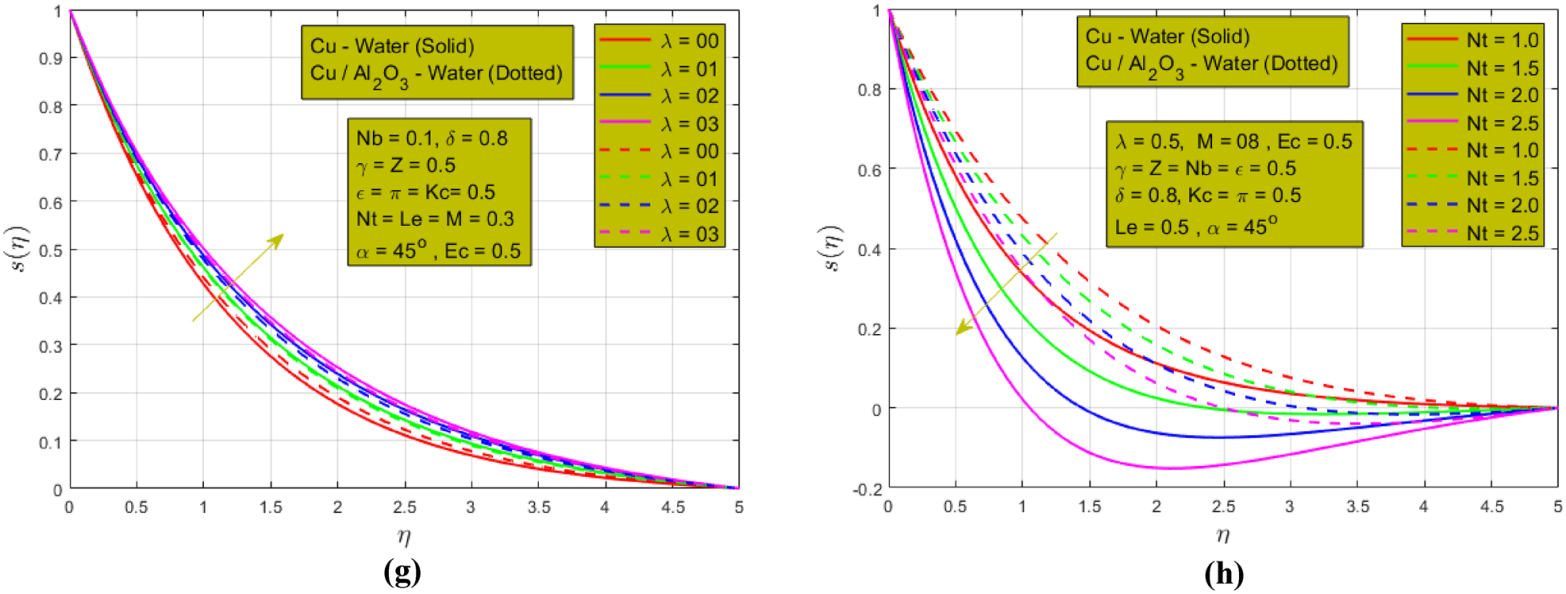
(**a**) Effect of $M$ on temperature $r(\eta )$. (**b**) Effect of $\lambda$ on temperature $r(\eta )$. (**c**) Effect of $\pi$ on temperature r $(\eta )$. (**d**) Effect of $Ec$ on temperature $r(\eta )$. (**e**) Effect of $\epsilon$ on concentration s $(\eta )$. (**f**) Effect of $Kc$ on concentration s $(\eta )$. (**g**) Effect of $\lambda$ on concentration s $(\eta )$. (**h**) Effect of $Nt$ on concentration s $(\eta )$.

The following Fig. 5a–h show the impression of different constraints on temperature and concentration profiles. The influence of magnetic parameters on temperature $r(\eta )$ is represented in Fig. 5a $\mathrm{when }Nb=0.1,\delta =0.8, \gamma =Z=\epsilon =\pi =Kc=Ec=0.5, Nt=Le=\lambda =0.3, \alpha =45^\circ$ and can be seen that the temperature increases when magnetic strength increases. The justification for this is that Lorentz force reduces the fluid motion and as a result, a maximum HT and a higher thermal BL are noted for HNF as compared to NF. Figure 5b indicates the effect of rotation on the temperature $(\eta )$ $\mathrm{when }Nb=0.1,\delta =0.8, M=0.3, Z=\epsilon =\pi =Kc=Ec=0.5,Nt=Le=\lambda =0.3, \alpha =45^\circ$. When the rotation of fluids increases the temperature profile shows the same behavior. Rotation increases temperature transmission by promoting fluid mixing, reducing boundary layer thickness, and enhancing convective heat transfer due to the formation of vortices and turbulence, leading to improved thermal. In this figure low but consistent HTR is noted for HNF. The influence of thermal radiation on the temperature $r(\eta )$ is shown in Fig. 5c $\mathrm{when }Nb=0.1,\delta =0.8, M=03, \gamma =Z=\epsilon =Kc=Ec=0.5, Nt=Le=\lambda =0.3, \alpha =45^\circ$. In the absence of the radiation parameter, a higher temperature BL is detected for both NFs. Thermal radiation typically decays temperature profiles by emitting heat energy from hotter regions to cooler surroundings, causing temperature gradients to diminish, resulting in a more uniform temperature distribution. The Eckert number characterizes the thermal energy conversion associated with the fluid’s motion. A higher Eckert number implies more kinetic energy, which promotes cooling, leading to a decrease in temperature gradients and a more uniform temperature profile. This phenomenon is presented in Fig. 5d $\mathrm{when }Nb=0.1,\delta =0.8, \gamma =Z=\epsilon =\pi =Kc=0.5,Nt=Le=\lambda =0.3, \alpha =45^\circ , M=03.$ However, we found that as viscous dissipation rises, the thermal BL gets thicker. The impact of mixed convection and CR is presented in Fig. 5e,f respectively. $\mathrm{When }Nb=0.1, M=08, \delta =0.8, \gamma =Z=\pi =Kc=Ec=0.5, Nt=Le=\lambda =0.3, \alpha =45^\circ$ the mixed convection and chemical reactions $\mathrm{when }Nb=0.1,\delta =0.8, \gamma =Z=\epsilon =\pi =Ec=0.5, Nt=Le=\lambda =0.3, M=08, \alpha =45^\circ$ decay concentration profiles due to enhanced fluid mixing from buoyancy and forced convection, leading to reduced concentration gradients and more uniform distribution. A higher concentration of BL is noted in the absence of mixed convection and CR for HNF as compared to NF which is presented in Fig. 5e,f for HNF. The rotation influence is represented in Fig. 5g $\mathrm{when }Nb=0.1,\delta =0.8, \gamma =Z=\epsilon =\pi =Kc=Ec=0.5, Nt=Le=M=0.3, \alpha =45^\circ$. The rotation and concentration profile have a direct relation. As the rotation parameter increases the mass transfer rate also increases. Rotation increases the concentration profiles due to the centrifugal forces that drive fluid motion. The thermophoresis parameter effect on the concentration profile is shown in Fig. 5h $\mathrm{when }Nb=0.1,\delta =0.8, \gamma =Z=\epsilon =\pi =Kc=Ec=0.5, M=08, Le=\lambda =0.3, \alpha =45^\circ$. A wider concentration of BL is observed for NF as compared to HNF when the thermophoresis parameter increases. This is due to the inclusion of single and double NPs inclusion in the host fluid. So, HNF shows a consistent mass transfer rate in Fig. 5h.

### Numerical outcomes

The numerical outputs for skin frictions ${Cf}_{x}$, ${Cf}_{y}$, Nusselt number ${Nu}_{x}$, and Sherwood number ${Sh}_{x}$ are presented in this section. Table 5 represents the obtained numerical results for skin frictions ${Cf}_{x}$ and ${Cf}_{y}$ for both nanofluids. A minimum skin friction ${Cf}_{x}$ is observed in the absence of rotation i.e. λ = 0 and increasing behavior is seen for increasing values of rotation parameter from $\lambda =1$ to $\lambda =2,$ while an opposite behavior is observed for ${Cf}_{y}.$ Increased porosity from $10\le Z\le 30$ reduces skin friction in porous media because higher permeability allows fluid to flow more easily through the medium, reducing the resistance to motion and minimizing the friction between the fluid and the porous structure. The stretching ratio is defined by the rate along the *y*-$axis$ to the stretching rate *x*-$axis$. Increased stretching ratio $0.9\le \gamma \le 1.1$ increases skin friction in the context of flow over a solid surface. This occurs because a higher stretching ratio leads to increased shear stress. Reduced skin friction is followed in the deprivation of mixed convection and magnetic field and increasing behavior is noted when it increases. The angle of inclination has a great influence on skin friction. When the magnetic field is parallel to the *x*-$axis$, a maximum skin friction ${Cf}_{x}$ is noted while it decays when the angle of inclination increases between the range $0\le \alpha \le 90.$ Minimum skin friction is noted when the magnetic field becomes parallel to the $y-axis.$ Table 6 shows the influences of different constraints on Nusselt number ${Nu}_{x}$ and Sherwood number ${Sh}_{x}$ for both nanofluids. A higher HMT rate is noted when the angle of inclination for the magnetic field is parallel to the *x*-$axis.$ Increasing radiation parameter $0\le \pi \le 4$ typically decreases the Nusselt and Sherwood numbers because radiative heat or mass transfer is a slower process compared to convection. As radiation becomes more significant, it diminishes the relative contribution of convection, leading to lower Nusselt and Sherwood numbers, indicating reduced HMTR and utmost Nusselt and Sherwood is seen in the absence of radiation, i.e. $\pi =0$. Prandtl number $7.2\le Pr\le 9.2$ and magnetic field $0\le M\le 15$ have a direct relation and inverse relation with HMTR respectively because $Pr$ implies that thermal or mass diffusion is rapid relative to momentum diffusion and the existence of magnetic field suppresses fluid movement, disrupting convection resulting in reducing HMTR. Increasing values of the CR parameter $0\le Kc\le 4$ and Lewis number $10\le Le\le 20$ decreases the Nusselt number while it increases the Sherwood number. CR consumes the temperature and concentration gradient and a higher Lewis number results in a more dominant effect on fluid behavior. Similar results are found for thermophoresis $0\le {N}_{t}\le 6$ and Brownian motion $0.5 \le {N}_{b}\le 1.5$ factors corresponding to the Nusselt number and Sherwood number. As the viscous dissipation parameter increases between $5\le Ec\le 15$ the HMT rate increases in both cases of used nanofluids because increased $Ec$ number indicates that the kinetic energy of the fluid is relatively higher than internal energy change.

**Table 5:** Numerical results for skin frictions along $x-axis$ $C{f}_{x}$ and $y-axis C{f}_{y}$ when $\pi =0.7, Pr=6.2, M=0.8, Le=0.6, Nt=0.3, Ec=0.5, Nb=0.2$ respectively.

$\lambda$	Z	$\gamma$	$\epsilon$	$M$	$\alpha$	$C{f}_{x}$ for NF	$C{f}_{y}$ for NF	$C{f}_{x}$ for HNF	$C{f}_{y}for$ HNF
$0.0$	$0.7$	$0.5$	$0.3$	$0.8$	45°	$-2.14850$	$-0.74840$	$-3.08458$	$-1.38589$
$1.0$						$-1.92663$	$-1.73975$	$-2.95226$	$-1.84510$
$2.0$						$-1.90891$	$-2.63017$	$-2.86364$	$-2.30971$
$0.5$	$10$	$0.5$	$0.3$	$0.8$	45°	$-4.28186$	$-2.23697$	$-4.37705$	$-2.25723$
	$20$					$-5.83925$	$-2.98470$	$-5.48274$	$-2.79401$
	$30$					$-7.06607$	$-3.58458$	$-6.40189$	$-3.24491$
$0.5$	$0.7$	$0.9$	$0.3$	$0.8$	45°	$-1.87550$	$-2.00135$	$-2.95067$	$-2.80315$
		$1.0$				$-1.86189$	$-2.20911$	$-2.94398$	$-3.11017$
		$1.1$				$-1.84833$	$-2.42367$	$-2.93726$	$-3.42038$
$0.5$	$0.7$	$0.5$	$0.0$	$0.8$	45°	$-2.12530$	$-1.40129$	$-3.08560$	$-1.70111$
			$01$			$-1.74097$	$-1.03913$	$-2.86990$	$-1.49433$
			$02$			$-1.38247$	$-0.69217$	$-2.65904$	$-1.29093$
$0.5$	$0.7$	$0.5$	$0.3$	$00$	45°	$-1.36554$	$-1.11913$	$-1.16233$	$-0.95291$
				$50$		$-9.76931$	$-4.93298$	$-17.8269$	$-8.93220$
				$10$		$-19.4546$	$-9.75085$	$-35.6205$	$-17.8196$
$0.5$	$0.7$	$0.5$	$0.3$	$0.8$	0°	$-1.36554$	$-1.11913$	$-1.16233$	$-0.95291$
					30°	$-1.70734$	$-1.19708$	$-2.26901$	$-1.31224$
					45°	$-2.00639$	$-1.29060$	$-3.02022$	$-1.63860$
					60°	$-2.27259$	$-1.38630$	$-3.62387$	$-1.91633$
					90°	$-2.51397$	$-1.48003$	$-4.14240$	$-2.16088$

**Table 6:** Numerical results for Nusselt number ${Nu}_{x}$ and Sherwood number ${Sh}_{x}$ when $\lambda =0.5, Z=0.7, \gamma =0.5, \epsilon =0.3, M=0.8$ respectively.

$\alpha$	$\pi$	$Pr$	$M$	$Kc$	$Le$	$Nt$	$Ec$	$Nb$	${Nu}_{x}$ for NF	${Sh}_{x}$ for NF	${Nu}_{x}$ for HNF	${Sh}_{x}for$ HNF
0°	$0.7$	$6.2$	$0.8$	$1.5$	$0.6$	$0.3$	$0.5$	$0.2$	$3.00058$	$1.72922$	$4.36870$	$1.74389$
30°									$2.74390$	$1.71586$	$3.04228$	$1.68995$
45°									$2.52236$	$1.70378$	$2.14219$	$1.65535$
60°									$2.32647$	$1.69307$	$1.42823$	$1.62959$
90°									$2.14992$	$1.68352$	$0.82732$	$1.60887$
45°	$00$	$6.2$	$0.8$	$1.5$	$0.6$	$0.3$	$0.5$	$0.2$	$10.1829$	$1.72429$	$6.59213$	$1.66263$
	$02$								$5.60675$	$1.68505$	$4.41799$	$1.64535$
	$04$								$4.05846$	$1.67249$	$3.37044$	$1.63681$
45°	$0.7$	$7.2$	$0.8$	$1.5$	$0.6$	$0.3$	$0.5$	$0.2$	$2.74899$	$1.70996$	$2.14219$	$1.65535$
		$8.2$							$2.98656$	$1.71649$	$2.44237$	$1.66191$
		$9.2$							$3.21141$	$1.72272$	$2.75913$	$1.66886$
45°	$0.7$	$6.2$	$00$	$1.5$	$0.6$	$0.3$	$0.5$	$0.2$	$3.00058$	$1.72922$	$4.36870$	$1.74389$
			$05$						$-2.2238$	$1.48799$	$-11.973$	$1.25456$
			$10$						$-6.6810$	$1.33243$	$-26.824$	$0.903258$
45°	$0.7$	$6.2$	$0.8$	$00$	$0.6$	$0.3$	$0.5$	$0.2$	$2.62898$	$0.58260$	$2.29389$	$0.504837$
				$02$					$2.49564$	$2.16376$	$2.10901$	$2.123040$
				$04$					$2.43024$	$4.10204$	$2.03810$	$4.07362$
45°	$0.7$	$6.2$	$0.8$	$1.5$	$10$	$0.3$	$0.5$	$0.2$	$2.46683$	$2.51915$	$2.07947$	$2.41901$
					$15$				$2.45546$	$2.75390$	$2.06659$	$2.64903$
					$20$				$2.44728$	$2.94512$	$2.05751$	$2.83747$
45°	$0.7$	$6.2$	$0.8$	$1.5$	$0.6$	$00$	$0.5$	$0.2$	$2.98666$	$1.64205$	$2.77205$	$1.61567$
						$03$			$0.487884$	$1.66930$	$-0.2628$	$1.46950$
						$06$			$0.060626$	$1.45236$	$-0.6053$	$1.16859$
45°	$0.7$	$6.2$	$0.8$	$1.5$	$0.6$	$0.3$	$05$	$0.2$	$6.268410$	$1.80742$	$11.6602$	$1.86675$
							$10$		$10.26151$	$1.91756$	$20.8553$	$2.07063$
							$15$		$18.72051$	$2.13910$	$28.9276$	$2.24937$
45°	$0.7$	$6.2$	$0.8$	$1.5$	$0.6$	$0.3$	$0.5$	$0.5$	$2.513160$	$1.79520$	$2.13120$	$1.71391$
								$1.0$	$2.497920$	$1.94580$	$2.11298$	$1.80989$
								$1.5$	$2.482801$	$2.09423$	$2.09489$	$1.90382$

## Conclusion

In this present article, the authors discussed the heat and mass transfer rate of 3D rotating, incompressible NF and HNF fluid over the dual stretchable surface. The governing equations are transformed and tackled in MATLAB. We considered the inclined magnetic field, viscous dissipation, thermal radiation, and chemical reaction effects to provide a theoretical view of the study. The main findings that one needs to remember from this study are stated below:

1. Increasing the rotation, magnetic force, and porosity of the SS decays the velocity profile while it increases by a rise in mixed convection and stretching ratio parameters.
2. The temperature profile $r(\eta )$ increases by an increase in rotation and magnetic field parameters.
3. The concentration profile decreases under the increasing influence of mixed convection parameters, and thermophoresis parameters whereas it increases by raising the value of the rotation parameter.
4. A maximum ${Nu}_{x }=3.00$ and $4.36$, ${Sh}_{x }=1.72$ and $1.74,$ and minimum ${Cf}_{x }=-1.36$ and $-1.16$, ${Cf}_{y}=-1.11$ and $-0.95$ is noted when $\alpha ={0}^{0}$ i.e., angle of inclination is parallel to the $x-axis,$ for NF and HNF.
5. By increasing Eckert number i.e., $5\le Ec\le 10,$ the ${Nu}_{x }=18.7, 28.9$, and ${Sh}_{x }=2.1, 2.2$ is noted for NF and HNF respectively.
6. When the chemical reaction parameter increases i.e., $0\le Kc\le 4,$ the Nusselt number $2.62\le {Nu}_{x }\le 2.43$ and $2.29\le {Nu}_{x }\le 2.03$ decreases, and Sherwood number ${0.58\le Sh}_{x }\le 4.10$ and $0.50\le {Sh}_{x }\le 4.07$ increases for NH and HNF respectively.
7. By changing the angle of inclination, the ${Nu}_{x}$ performance is noted at 8% for NF and 33% for HNF which proves the high heat transfer rate efficiency of HNF.

The strength of this study lies in its comprehensive analysis of the three-dimensional mixed convection flow of nanofluid over a dual stretching sheet, considering influential factors such as viscous dissipation, Joule heating, and solar radiation. The practical relevance to solar energy systems adds significance to the findings. However, limitations include potential simplifications in assumptions, applicability restricted to similar geometries, assumptions about material properties, and the influence of numerical solution techniques, emphasizing the need for careful interpretation of results in specific contexts.

## Data Availability

All data generated or analyzed during this study are included in this published article.

## List of symbols

$${\omega }^{*}$$: Angular velocity
$${C}_{p}$$: Specific heat
${\phi }_{1},{\phi }_{2}$: Volume fraction for nanoparticles
${G}_{1}, {G}_{2},{G}_{3}, {G}_{4}$: Constants for nanofluid
$T,C$: Temperature and concentration
$$Z$$: Porosity parameter
$a,b$: Stretching rate along x and y axis
$$Pr$$: Prandtl number
$\pi$: Thermal radiation parameter
$$\alpha$$: Inclination angle
$${B}_{0}$$: Magnetic field
${N}_{t}, {N}_{b}$: Thermophoresis and Brownian motion parameter
$${q}_{w}$$: Heat flux
*R*: Reynolds number
*Kc*: Chemical reaction
$${g}^{*}$$: Gravitational acceleration
$${\alpha }_{hnf}$$: Thermal diffusivity
$$\uplambda$$: Rotational velocity
$$\rho$$: Density
$\gamma$: Stretching ratio parameter
$$k$$: Thermal conductivity
${Nu}_{x},{Sh}_{x}$: Nusselt and Sherwood number coefficient
$$\eta$$: Similarity variable
$u,v$*, w*: Velocity components in ${\text{x,y,z}}$ direction
$${Cf}_{x}, {Cf}_{y}$$: Skin frictions
$${p}{\prime}, {q}{\prime}$$: Dimensionless velocity
${H}_{1}, {H}_{2},{H}_{3}, {H}_{4}$: Constants for hybrid nanofluid
*Sc*: Schmidt number
$${D}_{B}$$: Brownian diffusion
$${\beta }_{t}$$: Thermal expansion
$${v}_{f}$$: Kinematic viscosity
$${\mu }_{f}$$: Dynamic viscosity
$r,s$: Temperature and concentration profile
3D: Three dimensional
$$\sigma$$: Electrical conductivity
$${\tau }_{w}$$: Shear stress
$\epsilon$: Mixed convection parameter
